# In the Marathon toward Achieving Efficient Enzymatic Decarboxylation of Fatty Acids

**DOI:** 10.1021/acscentsci.5c02102

**Published:** 2025-11-12

**Authors:** Christoph K. Winkler, Wolfgang Kroutil

**Affiliations:** Institute of Chemistry, University of Graz, Heinrichstrasse 28, 8010 Graz, Austria

## Abstract

The turnover number for decarboxylation catalyzed by UndB was taken to the next level. Still 3 orders of magnitude to go for industrial applications.

## Graphical abstract

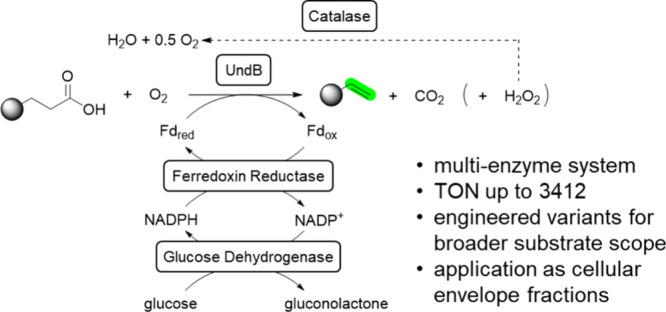

In this issue of *ACS Central Science*, Abhishek Sirohiwal, Debasis Das, and co-workers pushed an enzymatic approach for oxidative decarboxylation of fatty acids to obtain 1-alkenes to the next level.^1^ The production of 1-alkenes is of significant global interest due to their potential as green commodity chemicals and next-generation ‘drop-in’ biofuels. Thus, fatty acids derived from plant oils, animal fats, forestry, fermentation, algae, etc. could be converted to renewable-based aviation fuels, e.g., by decarboxylation. Chemical methods rely on high temperatures (180–340 °C), elevated pressures (8–40 bar), as well as metal catalysts and may lead to product mixtures, but have the significant advantage that they are run at molar substrate concentrations (see Table S1 in ref 2). Biocatalytic options^3^ may present an alternative, as such reactions are in general run at ambient temperature and pressure and may offer high selectivity, thereby minimizing side reactions.

The enzyme performing such a decarboxylation as investigated in the study discussed here (Scheme 1a)^1^ converted free C_*n*_ fatty acids into the corresponding C_*n*–1_ 1-alkenes and has been named UndB, originating from a *Pseudomonas* species.^4^ UndB is a nonheme di-iron-containing integral membrane enzyme requiring molecular O_2_ and an electron transfer system to provide the electrons needed. This indicates a complex system with several challenges, including (i) the use of a membrane enzyme, (ii) the requirement for molecular oxygen, (iii) the significant loss of reduction equivalents via uncoupled electron transfer from NADPH to O_2_, leading to the formation of hydrogen peroxide, (iv) the potential inhibition and enzyme inactivation by the side product hydrogen peroxide, and (v) the efficient supply of the required electrons. For the latter challenge, it was found that the ferredoxin and ferredoxin reductase from the cyanobacterial *S. elongatus* represent the most efficient system to provide the electrons. As the ferredoxin reductase requires NADPH as an electron source, the paper reports here the installation of a NADPH regeneration system based on glucose dehydrogenase and glucose as the reducing agent, a system broadly applied in biocatalysis. To address the challenges associated with using a membrane enzyme, cellular membrane envelope fractions (CEF, prepared by cell lysis and centrifugation) were successfully applied as the active catalyst preparation. To minimize the potential negative effects of H_2_O_2_ side product formation, a catalase was added to disproportionate H_2_O_2_ into water and O_2_. With these adaptations to the reaction system, a turnover number (TON) of 3412 was achieved for UndB using lauric acid C12:0 (1 mM) as the substrate representing an improvement to a recent report.^5^

**Scheme 1:**
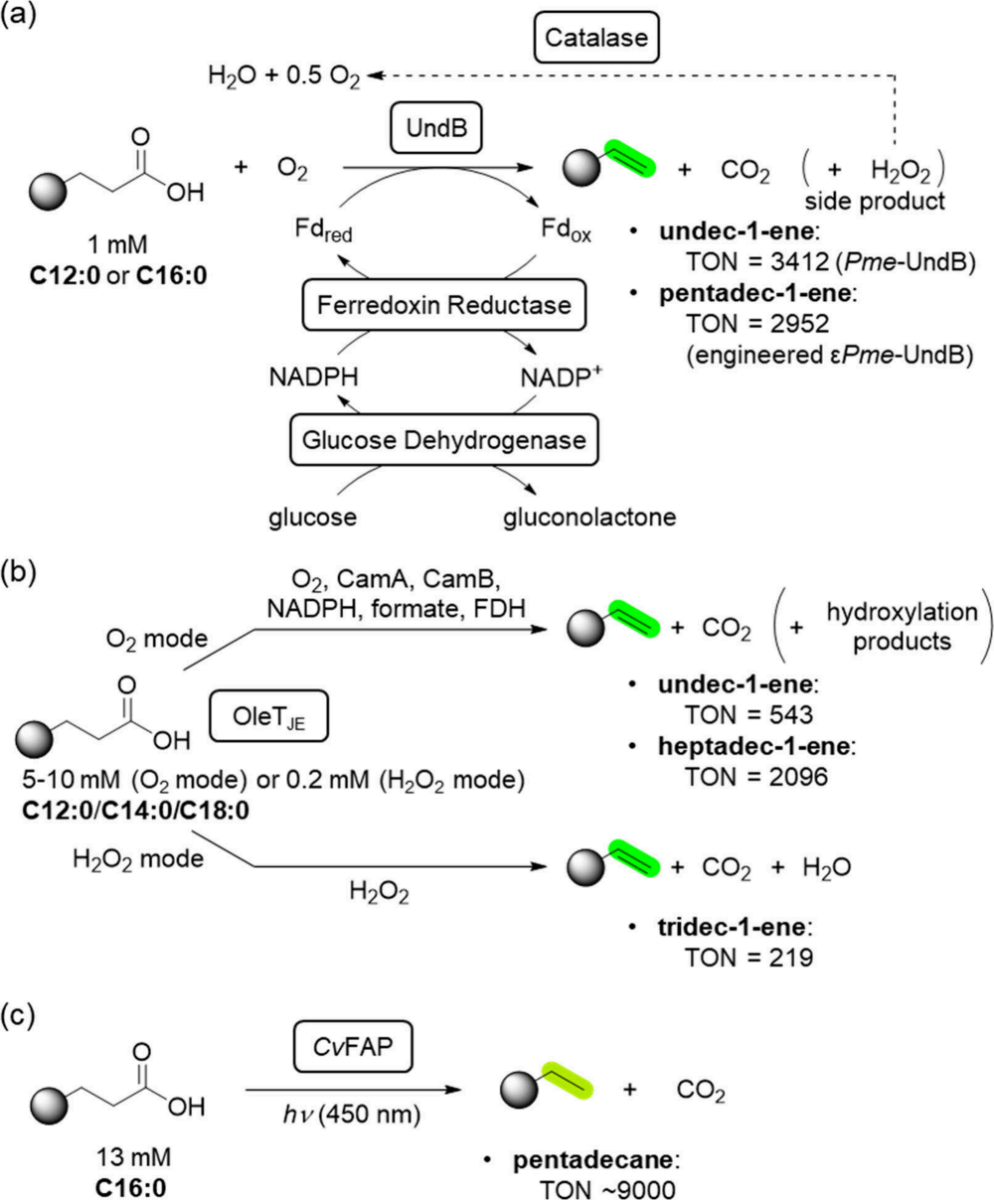
Comparison of Reaction Concepts for the Enzymatic Decarboxylation of Fatty Acids^a^

a (a) UndB-catalyzed reaction. (b) Two different pathways for the decarboxylation of fatty acids by the CYP152 OleT_JE_, following either a very similar concept as in (a) (O_2_ mode) or using H_2_O_2_ as the oxidant (H_2_O_2_ mode). (c) Light-dependent redox-neutral decarboxylation of fatty acids using the fatty acid photodecarboxylase *Cv*FAP. Fd = ferredoxin; CamA and CamB = putidaredoxin/putidaredoxin reductase system; FDH = formate dehydrogenase.

This represents a 262-fold improvement compared to a previous study in which UndB was used alone and a 13-fold improvement over the best previous report involving a catalase fused to UndB (TON 271).

Based on the orientation of certain helices of the AlphaFold2 structures of a range of related enzymes, the authors clustered the enzyme family into two classes and noticed differences in their substrate preferences. While class I enzymes, such as UndB, preferred lauric acid C12:0 as the substrate, class II members preferentially decarboxylated palmitic acid C16:0. Remarkably, this specificity could be correlated with a distal region of the enzyme (residues 154–184 in UndB) and the TON for palmitic acid C16:0 could be improved by exchanging these residues for the equivalent region in the orthologue from *Paraglaciecola psychrophile* (from a TON of 1152 to a TON of 2952). The effect of this region on substrate specificity was rationalized with MD simulations and traced back to volumetric modulation of the substrate binding pocket.

A limitation of the proposed UndB-based system at the current stage is the low substrate concentration (1 mM), which is 3 orders of magnitude below the concentrations used in chemical approaches. Comparing it with other enzymatic approaches, soluble heme-dependent enzymes, such as the CYP152 group, need to be mentioned, as they can perform both decarboxylation and hydroxylation reactions.^6^ One CYP152 that preferentially decarboxylates fatty acids is OleT_JE_, which can be driven in different modes. The first mode is similar to that described above for UndB, where the reaction system also comprises an electron transfer system (putidaredoxin/putidaredoxin reductase CamAB as surrogate redox partners) and formate or phosphite dehydrogenase for NADPH regeneration (Scheme 1b, O_2_ mode). This approach allowed the decarboxylation of, e.g., lauric acid C12:0 at 10 mM concentration, giving 3.26 mM of alkene (TON ≈ 500), or of stearic acid (C18:0) at 5 mM substrate conc., reaching a TON of 2000.^7^ From this point of view, the current work achieves a similar TON range for UndB.

On the other hand, OleT_JE_ can also be used with H_2_O_2_ as the only oxidant, circumventing the need for the electron transport chain, as well as the need for O_2_ and any reducing agent (Scheme 1b, H_2_O_2_ mode). This approach would be favored from a practical point of view, as only a single enzyme is required; however, the TON requires further improvement, as the highest number reported is 194 for myristic acid C14:0 at a 0.2 mM substrate concentration.^8^ If drop-in biofuels are the target, the transformation of fatty acids such as C12–C20 to saturated alkanes can be considered as well.^9^ For this purpose the flavin dependent fatty acid photodecarboxylase (FAP) requires only light without the need for any external electron source for cofactor regeneration, reaching TONs up to 9000 at a 13 mM substrate concentration (Scheme 1c).^10^ Due to the radical mechanism within a shielded active site, side reactions with saturated fatty acids have not been observed; however, the inherent photolability of the enzyme is limiting.

To be of interest for bulk applications, ideally the turnover number should be around 800,000, the reaction should be independent of sacrificial reagents for cofactor recycling to reduce costs, and compatible with high substrate concentrations.^11^ For this system to become a viable option for industry,^12^ there are still improvements of 3 orders of magnitude in TON, as well as increases in substrate concentration and improved atom economy required.

In this paper, an important step has been taken to increase the TON for UndB-catalyzed decarboxylation into the 10^3^ range to potentially develop a viable enzyme-based method to convert fatty acids into hydrocarbon biofuels as an eco-friendly alternative compared to current chemical technologies.
